# Genome-wide analyses reveal footprints of divergent selection and popping-related traits in CIMMYT’s maize inbred lines

**DOI:** 10.1093/jxb/eraa480

**Published:** 2020-10-18

**Authors:** Jing Li, Delin Li, Cristian Zavala Espinosa, Viridiana Trejo Pastor, Awais Rasheed, Natalia Palacios Rojas, Jiankang Wang, Amalio Santacruz Varela, Natália Carolina de Almeida Silva, Patrick S Schnable, Denise E Costich, Huihui Li

**Affiliations:** 1 Institute of Crop Sciences, The National Key Facility for Crop Gene Resources and Genetic Improvement and CIMMYT China office, Chinese Academy of Agricultural Sciences, Beijing, China; 2 Data Biotech (Beijing) Co., Ltd., Beijing, China; 3 Department of Plant Genetics and Breeding, China Agricultural University, Beijing, China; 4 International Maize and Wheat Improvement Center (CIMMYT), Texcoco, Mexico; 5 Colegio de Postgraduados, Km 36.5, Carretera México-Texcoco, Montecillo, México; 6 Department of Plant Sciences, Quaid-i-Azam University, Islamabad, Pakistan; 7 Universidad Tecnológica del Uruguay, Durazno, Uruguay; 8 Data2Bio LLC, Ames, USA; 9 Department of Agronomy, Iowa State University, Ames, IA, USA; 10 University of Trento, Italy

**Keywords:** EigenGWAS, GWAS, maize adaptation, popping traits, quality traits, tropical maize landrace

## Abstract

Popcorn (*Zea mays* L. var. *Everta*) is the most ancient type of cultivated maize. However, there is little known about the genetics of popping-related traits based on genotyping-by-sequencing (GBS) technology. Here, we characterized the phenotypic variation for seven popping-related traits in maize kernels among 526 CIMMYT inbred lines (CMLs). In total, 155 083 high-quality single nucleotide polymorphism (SNP) markers were identified by a GBS approach. Several trait-associated loci were detected by genome-wide association study for color, popping expansion volume, shape, pericarp, flotation index, floury/vitreous, and protein content, explaining a majority of the observed phenotypic variance, and these were validated by a diverse panel comprising 764 tropical landrace accessions. Sixty two of the identified loci were recognized to have undergone selection. On average, there was a 55.27% frequency for alleles that promote popping in CMLs. Our work not only pinpoints previously unknown loci for popping-related traits, but also reveals that many of these loci have undergone selection. Beyond establishing a new benchmark for the genetics of popcorn, our study provides a foundation for gene discovery and breeding. It also presents evidence to investigate the role of a gradual loss of popping ability as a by-product of diversification of culinary uses throughout the evolution of teosinte–to–modern maize.

## Teaser

Sequencing-based technology reveals popping characteristics in maize, establishing a benchmark for popcorn genetics, and supporting a gradual selection process for popping traits.

## Introduction

Maize originated in Mexico about 9000 years ago (Matsuoka *et al.*, 2002; Piperno *et al.*, 2009). Maize landraces named “ancient indigenous” include Chapalote, Palomero Toluqueño, Arrocillo and Nal-Tel, all originating from Mexico, directly from Teosinte (*Zea* species), and are known for their ability to pop (Wellhausen *et al.*, 1952). Since the earliest wild and cultivated forms of maize are all popcorn types, popcorn (*Zea mays* L. var. *Everta*) from Mexico is considered to be the most ancient maize type, and its genome sequence should contain the most comprehensive sample of genomic variation of maize (Vielle-Calzada *et al.*, 2009). In 2009, Mexican scientists sequenced the popcorn landrace called Palomero Toluqueño (popcorn from the Toluca area, west of Mexico City, in the state of Mexico) (Vielle-Calzada *et al.*, 2009), but these data could not be used to study popcorn genome characteristics and structural variation, due to the short contigs generated by the sequencing technology available at that time. Therefore, to our knowledge, there has been no genomic characterization of popcorn until now.

The Maize HapMap (version 2) shows that genomic structural variation is enriched near the regions associated with traits (Suo *et al.*, 2012; Liu *et al.*, 2016a). Thus, the discovery of quantitative trait loci (QTL) by genome-wide association analysis is not only beneficial for the analysis of the genetic structure of complex traits, but also for the in-depth study of genomic structural variation (Huang *et al.*, 2010). Still, many questions about the process of maize domestication remain unclear because modern maize specimens do not represent the full range of past diversity. This is due to the abandonment of unproductive landraces, genetic drift, on-going natural selection, and recent breeding activities. The complex evolutionary history of maize (*Zea mays* L. ssp. *mays;*Matsuoka *et al.*, 2002; Chia *et al.*, 2012; Kistler *et al.*, 2018) still needs to be clarified by studies of genetic diversity in materials that bridge the gap between teosinte and modern maize. This could include materials such as archaeological cob remains from different stages in the domestication process (Ramos-Madrigal *et al.*, 2016), as well as the most ancient types of maize (Vielle-Calzada *et al.*, 2009). 

Popcorn plays a key role in the history and spread of maize, so it is critical to understand the genetic basis of an ancestral trait such as popping, which is shared with the crop’s closest common ancestor. However this trait was subsequently lost, as other kernel types were selected in Mexico during domestication, primarily for diverse culinary uses.

Currently, there are several methods for phenotyping popping-related traits in popcorn, as well as other races of maize with similar properties. Popping volume, defined as the popped volume per 100 kernels, is the most important popping-related trait, which has been measured in all popping-related publications (Robbins and Ashman, 1984; Dofing *et al.*, 1990, 1991; Daros *et al.*, 2002; Lu *et al.*, 2003; Babu *et al.*, 2006; Li *et al.*, 2007a, 2008, 2009). In addition, flake size, popping rate, percentage of unpopped expansion (defined as the flake volume per popped kernel), the ratio of popped versus unpopped kernels, and the number of unpopped kernels per 200 kernels after popping, were evaluated in a few studies (Babu *et al.*, 2006; Li *et al.*, 2007a, 2008, 2009). Even though there has been considerable work on popping characteristics, the different measurement conditions and standards have led to conflicting results and only served to increase the difficulty of further research. Some of the methods employed a specific amount of vegetable or animal oil to facilitate the cooking of the popcorn kernels in containers. Other methods utilized a microwave oven, simulating the way popcorn is cooked in small grocery stores, fast food restaurants, homes, etc. For example, different sample quantities of kernels (weights and numbers of kernels) were chosen to measure popping volume, including 40 g (Daros *et al.*, 2002), 75 g (Robbins and Ashman, 1984; Lu *et al.*, 2003), 100 kernels (Li *et al.*, 2007a, 2008, 2009), 150 g (Dofing *et al.*, 1991), and 200 kernels (Babu *et al.*, 2006). Different popping machines were also used, combined with different treatments: (i) Cretors 1100-W popper (Cretors Co., Chicago, IL) using 50 g of partially hydrogenated soybean oil (Dofing *et al.*, 1990, 1991) or 25 ml peanut oil (Robbins and Ashman, 1984; Lu *et al.*, 2003) to pop each sample; (ii) BZ-99 popping machine (Shanghai Duoli Food machine building company, Shanghai, China; Li *et al.*, 2007a, 2008, 2009); and (iii) microwave ovens with a variety of different powers, a 700 W Litton (Dofing *et al.*, 1990) and 900 W power (Daros *et al.*, 2002; Babu *et al.*, 2006). A comprehensive dissection of popping characteristics would require that a standard and widely accepted popping measurement protocol be developed.

The genetic variation of popcorn has been studied using bi-parental populations and simple sequence repeat (SSR) markers (Babu *et al.*, 2006; Li *et al.*, 2007a, 2007b, 2008, 2009; see Supplementary Table S1 at *JXB* online). In these studies, both the restricted diversity of the parents and low marker density limited a comprehensive dissection of popcorn genetic variation. Current sequencing technologies are both high throughput and highly accurate, thus providing an opportunity to more thoroughly dissect the popcorn genome, and reveal the genomic variation that underlies the evolution and domestication of maize. Here we apply conventional genotyping-by-sequencing (cGBS), which is a technically simple, highly multiplexed approach (Elshire *et al.*, 2011) that has been applied to many crops (Poland *et al.*, 2012; Lu *et al.*, 2013; Morris *et al.*, 2013; Schmutz *et al.*, 2014), and used to study the genetic architectures of many phenotypic traits in maize (Riedelsheimer *et al.*, 2012; Romay *et al.*, 2013).

A number of studies have focused on popping volume and the genetic relationship between this popping characteristic and yield (Dofing *et al.*, 1991; Daros *et al.*, 2002; Babu *et al.*, 2006; Li *et al.*, 2008). However, efforts to unravel information on the ability of quality traits to contribute towards superior popping expansion volume have been very limited. Quality traits are critical to determine the basis for popping ability in popcorn. For example, starch and protein contents have been reported to greatly influence popping ability (Brunson, 1937; Haugh *et al.*, 1976; Robbins and Ashman, 1984; Da Silva *et al.*, 1993; Park and Maga, 2002). Zhou *et al.* (2016) analyzed the relationship between quality traits and popping ability by transferring the *O2* gene into two popcorn inbred lines: they found that the *o2* gene reduced the crude protein content and the expansion volume. Li *et al.* (2020) recently reported the genome sequence and annotation of a South African QPM line K0326Y, identifying a mutation of *o2* that suggests a potential role in vitreous endosperm formation. Vázquez-Carrillo *et al.* (2019) found a positive correlation between protein and expansion volume, a negative correlation between starch or floury/vitreous ratio and expansion volume, and a negative correlation between protein content and percentage of unpopped kernels and popped kernel size. These findings on popping quality traits are purely phenotypic correlations, and the specific genetic mechanism underlying these traits, for example, the number and map locations of the genes that affect popping quality characteristics throughout the genome, as well as the interactions among them, are still not well understood, and require further study.

Therefore, in this study, to characterize the genetic basis of popping-specific traits, we performed the following: (i) conducted GWAS on seven popping-related traits in 526 CIMMYT (The International Maize and Wheat Improvement Center) inbred lines (CMLs) using 155 083 high-quality cGBS SNPs; (ii) validated the expansion-volume-related loci in a diverse set of 764 tropical landrace populations; and (iii) tested if the loci associated with popping-related traits had undergone selection. The results of this study create a new resource for popcorn genetics to dissect the origin and evolution of maize.

## Materials and methods

### A panel of CIMMYT inbred lines (CMLs) and the genotyping platform

**Fig. 1.**
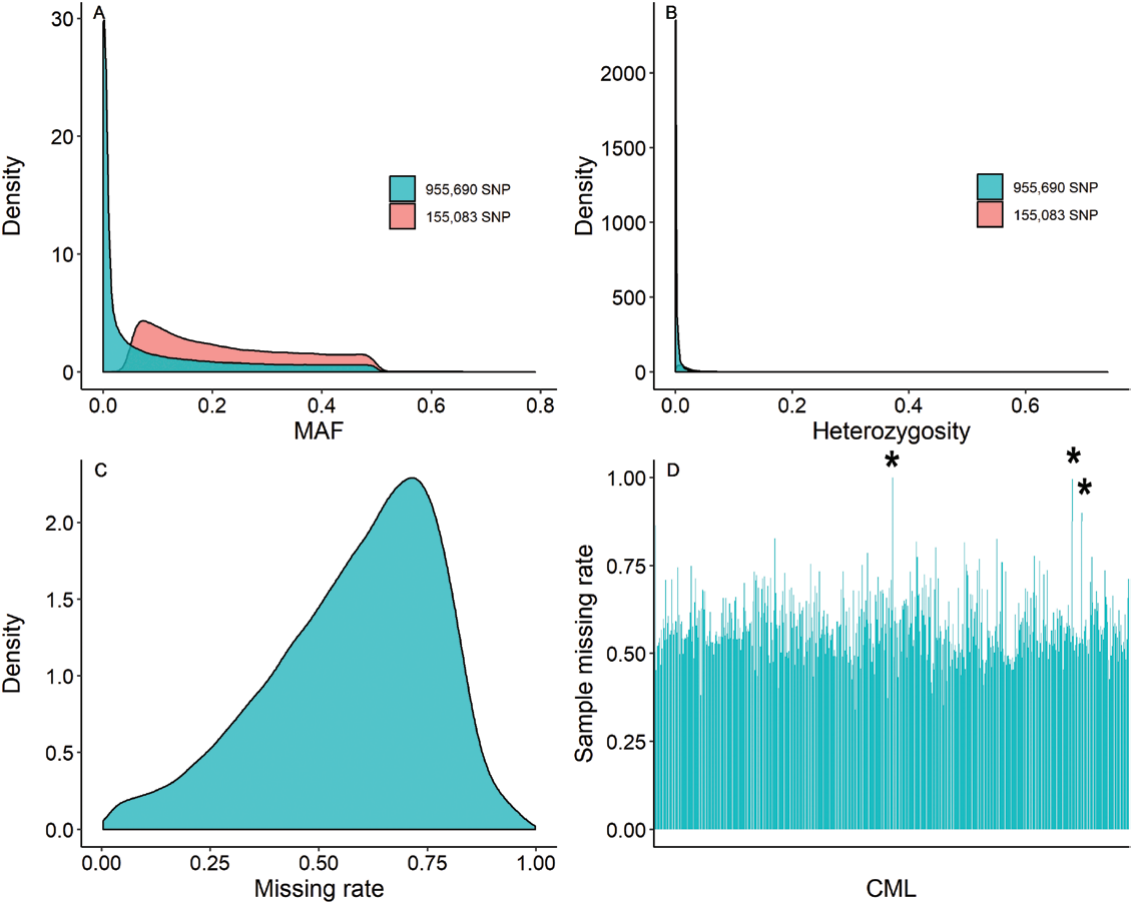
Basic information summarized from cGBS genotyping. Two data sets are used: unfiltered (blue) and filtered (pink). (A) SNP minor allele frequency; (B) SNP heterozygosity; (C) overall missing rate for 955 690 SNPs; (D) missing rate for each of the 529 CMLs (in numerical order), where asterisks indicate the three samples deleted from analysis due to high missing rate (i.e. CML342, CML529, and CML539).

A set of 529 CMLs released by CIMMYT’s Maize Breeding Program from 1960 to 2017 (http://hdl.handle.net/11529/10246) was used as an association mapping panel. Field experiments were conducted at Mexico, including those at Agua Fria, El Batan, HA, Palmira, RL, and Tlaltizapan, based on the adaptation groups. Each accession was planted in a single 1 m row with 30 cm spacing between rows (Supplementary Table S2). DNA was extracted from leaf tissues for each sample using the Cetyl Trimethyl Ammonium Bromide (CTAB) method (Murray and Thompson, 1980). This panel was genotyped for single nucleotide polymorphisms (SNPs) using conventional genotyping-by-sequencing (cGBS) at the Institute for Genomic Diversity, Cornell University, Ithaca, NY, USA (http://hdl.handle.net/11529/10423). In total, there were 955 690 SNPs retained, and their physical coordinates were derived from the maize reference genome version B73 AGPv2 (https://www.maizegdb.org/genome/genome_assembly/B73%20RefGen_v2). Imputation was performed with Beagle (V4.1; Browning and Browning, 2007, 2016), using the following parameters: window=50 000, overlap=3000, iterations=5, and clusters=0.005. From this, a smaller dataset of 155 083 SNPs that met the filtering criteria of call rate (CR)≥0.4, heterozygosity≤0.1, minor allele frequency (MAF)≥0.05, and Mendelian-transmitted markers was used for GWAS; three samples (CML342, CML529, and CML539) with a missing data rate ≥90% were removed (Fig. 1). Linkage disequilibrium decay was calculated using PopLDdecay (Zhang *et al.*, 2019) with the filtered dataset.

### Measurements of popping-related traits

The CMLs were planted at different locations in Mexico according to their adaptation, and the resulting seeds were harvested and deposited in the CIMMYT Germplasm Bank. For popping evaluations, the methods from two previous studies were used (Erazo-Barradas, 2009; Rangel *et al.*, 2011). A detailed description of the procedure for popping and phenotyping popcorn seed accessions stored in CIMMYT’s maize collection is available at http://hdl.handle.net/11529/10548274. The sample quantity was 30 g of kernels. Kernels with 13% moisture content were used, to promote gelatinization of the endosperm cells during the popping process, and to ensure that the maximum efficiency was obtained in expansion (Erazo-Barradas, 2009; Rangel *et al.*, 2011). The kernels were placed in a controlled environment of a germination chamber with the following conditions: 70% relative humidity and 21.1 °C for 10 d in order to reach the 13% moisture content (Hallauer, 2000). The samples were then popped individually using a microwave oven (110 V; output power 1000 W; consumed power 1400 W) set at 70% power for a cooking time of 2 min 45 s.

The predominant color of the popped kernels, denoted as color, was identified empirically and recorded as (1) cream or (2) white (Costich *et al.*, 2020). The popping expansion volume (PEV) refers to the absolute volume of 30 g of popped kernels, and was measured using a graduated cylinder (Erazo-Barradas, 2009; Costich *et al.*, 2020). The shape of the popped kernels, denoted as shape, was described with a qualitative 1–5 scale as follows: (1) mushroom or ball shape; (2) flake shape a little more extended (less rounded); (3) unilateral expansion; (4) bilateral expansion; and (5) multilateral or “butterfly”-shape (Costich *et al.*, 2020). The degree of pericarp retention, denoted as pericarp, was measured with a quantitative 1–5 scale, as follows: (1) popped kernels retaining between 81–100% of the pericarp, (2) 61–80% pericarp retention, (3) 41–60%, (4) 21–40%, and (5) 0–20% (Costich *et al.*, 2020) pericarp retention. Flotation index (IF) is the number of kernels that floated in a NaNO_3_ solution [ρ=1.250±0.001 (1.251 to 1.249) mg ml^-1^]. Floury/Vitreous was defined as the ratio of floury to vitreous endosperm types. Six kernels per replicate were randomly selected for scanning. The resulting images were analyzed and the areas of floury and vitreous endosperm were measured by WinSEEDLE (Regent Instruments Canada Inc.). As a result, the ratio of the area of floury versus vitreous endosperm was calculated for each kernel, and the average value was used for further analyses. The percentage of protein was evaluated using a near-infrared spectrometer (NIRs) FOSS 6500 (FOSS NIRSystems, Inc., Silver Spring, MD, USA). Spectra were collected between 400 nm and 2500 nm, registering the absorbance values log(1/R) at 4 nm intervals for each sample (Rosales *et al.*, 2011). These four popping specific traits (color, PEV, shape, and pericarp) and three quality traits (IF, floury/vitreous, and protein content) were measured at CIMMYT Headquarters (El Batán, Mexico) in the Maize Nutrition Laboratory “Evangelina Villegas”, with two replications (Table 1).

**Table 1.** Description of the seven kernel traits measured for this study.

Trait Name	Full Name	Description	Ranges, Units of Measurement
Color	Kernel Color	The predominant color of the popped kernels.	White, Cream
PEV	Popping Expansion Volume	The absolute volume of 30 grams of popped kernels.	ml
Shape	Popcorn Shape	The shape of popped kernels.	Scale of 1–5
Pericarp	Pericarp Retention	The amount of pericarp remaining after popping.	Scale of 1–5
IF	Flotation Index	The number of kernels that floated in a NaNO_3_ solution.	Scale of 1–5, 1: (0–12 kernels), 2: (13–37 kernels), 3: (38–62 kernels), 4: (63–87 kernels), 5: (88–100 kernels)
Floury/Vitreous	Floury/Vitreous	The ratio of floury to vitreous area in the endosperm.	Range is 0.28–4.01
Protein	Protein	The percentage of protein in the kernels.	Range is 8.61–16.48%

### Estimation of genotypic values and trait heritability

For all seven traits, best linear unbiased predictions (BLUPs) were calculated by fitting the following random model using the “lme4” package in R (De Boeck *et al.*, 2011):

$$\begin{array}{l} Y_{ij}=Entry_{i}+Rep_{j}+\varepsilon_{ij} \end{array}$$

where *Y*_*ij*_ is the trait observation for entry *i* in replication *j*; Entry_*i*_ is the random effect of entry *i*; *Rep*_*j*_ is the random effect of replication *j*; and *ε*_*ij*_ is the residual effect. The graphical display of trait correlation was done with the corrplot() function by Pearson correlation coefficient from the R package “corrplot” (Wei and Simko, 2013). Variance components, i.e. $\sigma_{G}^{2}$ and $\sigma_{\varepsilon }^{2}$ for genotype and residual effects were estimated from analysis of variance (ANOVA) with QTL IciMapping V4.2.23 (Meng *et al.*, 2015). Broad-sense heritability (*H*^2^) of each trait was estimated as:

$$\begin{array}{l} H^{2}=\sigma_{G}^{2}/\left(\sigma_{G}^{2}+\frac{\sigma_{\varepsilon }^{2}}{r}\right), \end{array}$$

where *r* is the number of replications (=2 in this study).

### Identification of genomic loci using Genome Wide Association Study (GWAS)

To minimize false positives and increase statistical power, population structure and cryptic relationships were considered. An iterative usage of *F*ixed *a*nd *r*andom *m*odel *C*irculating *P*robability *U*nification (FarmCPU; Liu *et al.*, 2016b) performed by *M*emory-efficient, *V*isualization-enhanced, and *P*arallel-accelerated Tool (MVP; https://github.com/XiaoleiLiuBio/MVP/), was used for the association analysis, where the first three principal component analysis (PCA) values (eigenvectors) were included as fixed effects in the mixed model to correct for stratification (Price *et al.*, 2006). To determine the cutoff level for declaring the significance of loci, the *P*-value threshold was determined using permutation tests with reshuffling of the PEV trait 1000 times. After log_10_ transformation, a –log_10_(*P*-value) threshold of 6.46 was used for an experimental type I error rate of 0.05. To balance the false positives and false negatives, the whole-genome –log_10_(*P*-value) significance cutoff was finally set at 5. The total phenotypic variation explained by all of the significant SNPs was estimated by the coefficient of determination *R*^2^ from multiple linear models using the “lm” function in R (Chambers and Hastie, 1992).

Functional annotations of the target SNPs were performed using SnpEff (Cingolani *et al*., 2012). The maize B73 reference V2 gene annotation was downloaded (as a gff3 file) from the Maize Genetics and Genomics Database (MaizeGDB; https://www.maizegdb.org/assembly). Based on the genome annotation, SNPs were categorized as being located in coding regions (i.e. overlapping with a coding exon), splice sites (within 2 bp of a splicing junction), 5′ UTRs and 3′ UTRs, intron, upstream and downstream regions, and intergenic regions. SNPs in coding regions were further grouped into synonymous SNPs (not causing amino acid changes) or non-synonymous SNPs (causing amino acid changes, including stop gain and stop loss). Functional enrichment analysis of the annotated genes was performed using the ClueGO plug-in for Cytoscape 3.6.1 (Bindea *et al.*, 2009).

### Identification of genomic loci that have undergone selection

Identification of potential selective signals during maize domestication and improvement through genome-wide association studies of eigenvectors was implemented using the bottom-up searching strategy EigenGWAS (Chen *et al.*, 2016), which is a single-marker regression approach based on principal component analysis. EigenGWAS identifies regions of the genome underlying population genetic differentiation in any genetic data where the underlying population structure is unknown, or where the interest is in assessing divergence along a gradient. Using 155 083 high-quality SNPs to generate a genetic relationship matrix, the top ten eigenvalues and their corresponding eigenvectors (i.e. Ev1 to Ev10) were calculated. SNP effects, nearly equivalent to fixation index (*F*_*st*_) (Wright, 1951), could be estimated by regressing each SNP for a selected eigenvector. To exclude the effect of genetic drift in selection loci mapping, the *p*-value was adjusted by a genomic control factor (Devlin & Roeder, 1999), and consequently the corrected *p*-value, *P*_*GC*_, was used for detecting the loci under selection_._ The SNPs with the top 5% of *P*_*GC*_ values (i.e. *P*_*GC*_<0.0266) were considered to have undergone selection.

### Validation of genomic loci in a panel of tropical landrace populations

A total of 764 heterogeneous and heterozygous tropical landraces originating from 20 countries was used in validation (Supplementary Table S3; Li *et al.*, 2019), all of which can be found in the CIMMYT Gemplasm Bank collection (http://mgb.cimmyt.org/gringlobal/search.aspx). The origins of these landraces represent diverse ecological regions including lowland tropical, sub-tropical/mid-altitude, and highland tropical subgroups, which were planted in Celaya and Tlaltizapan in 2016 (Table S3). Tunable genotyping-by-sequencing (tGBS^®^, Ott *et al.*, 2017) provided higher SNP calling accuracy, especially at heterozygous sites, with less missing data than conventional genotyping-by-sequencing (cGBS) with the same number of reads per sample (Ott *et al.*, 2017). This was achieved by two strategies: (i) two restriction enzymes were used in tGBS to digest the genomic DNA with overhangs in an opposite orientation, which ensures only double-digested fragments are amplified and sequenced; and (ii) additional adjustable selective nucleotides of primers were used in PCR amplification: this offers an additional genome reduction. The 12 selfed seeds of F_1_ from a single ear for each accession that was selected to be representative of the accession, were grown in the greenhouse, and the DNA from the seedlings bulked for tGBS sequencing in Data2Bio.

In total, 0.31 terabases (Tb) of sequence data from 2.5 billion quality-trimmed reads were generated via tGBS^®^ (Ott *et al.*, 2017). We identified 3 713 115 SNPs after alignment to the reference genome B73 AGPv3 (https://www.maizegdb.org/). Of the 3 713 115 tGBS SNPs, 65 540 were retained after filtering for MAF and heterozygosity. The 65 540 tGBS SNPs were imputed without a reference panel using Beagle (V4.1). To further capture the genetic variation across the maize genome, the 65 540 SNPs from tGBS were also imputed to 359 618 high-quality SNPs using maize HapMap V3 as a reference panel (https://www.maizegdb.org/genome/genome_assembly/B73%20RefGen_v3). Finally, the union of the two SNP sets (N=414 124 SNPs) was filtered by MAF and heterozygosity under the same criteria as aforementioned, resulting in 355 442 high-quality SNPs which were retained for validation.

To conduct tGBS, the restriction enzymes *Nsp*I and *Bfu*CI were used to digest the genomic DNA; while the single restriction enzyme *Ape*KI had been used to generate the cGBS data (Elshire *et al.*, 2011). Consequently, tGBS and cGBS identified very different sets of polymorphisms in the two datasets (CMLs versus tropical landraces). Because the CMLs were genotyped using the cGBS platform, and SNPs were called by B73 AGPv2, to validate the significant loci associated with the seven traits identified by CMLs, the physical positions of B73 AGPv2 were converted to those of B73 AGPv3 by http://ensembl.gramene.org/Oryza_sativa/Tools/AssemblyConverter?db=core. An extended haplotype homozygosity (EHH) test was conducted for the target SNPs within a 2 Mb region, identifying long and frequent haplotypes as implemented in the R package “rehh” (Gautier and Vitalis, 2012). Color, PEV, shape, and pericarp for each accession were measured at CIMMYT Headquarters (El Batán, Mexico), and were used to determine the extreme phenotypes, and to validate the QTLs identified in the CML population (Supplementary Table S3).

## Results

### Genotypic data analysis and population structure

The average MAF for the final selected dataset (*n*=355 442 SNPs) was 0.22 and the heterozygosity was 0.02. The heterozygosity of more than 98% of the SNPs was lower than 0.05 (Fig. 1A, B). The 155 083 SNPs are relatively evenly distributed across the 10 chromosomes (Supplementary Fig. S1), and average missing rate was 0.58. (Fig. 1C). In addition, the decay of linkage disequilibrium (LD) with physical distance between SNPs occurs at only 1.5 kb (decaying to *r*^2^ of 0.1) (Fig. S2). Principal component analysis revealed that there was a moderate population structure (Fig. S3): the panel included 30 tropical highland, 215 sub-tropical/mid-altitude, and 284 tropical lowland inbred lines, three samples with high missing rate were removed (Fig. 1D). Clear clustering based on adaptation was observed, while the tropical lowland group overlapped partially with the sub-tropical/mid-altitude group (Fig. S3). The tropical highland and tropical lowland populations were relatively scattered, indicating that there exists broad genetic variation within this set of 526 CMLs (Fig. S3). The passport information of 526 CMLs is shown in Table S2. Further information about these lines can be found at this site: https://data.cimmyt.org/dataset.xhtml?persistentId=hdl:11529/10246.

### Phenotypic correlation in the panel of CIMMYT inbred lines

**Fig. 2.**
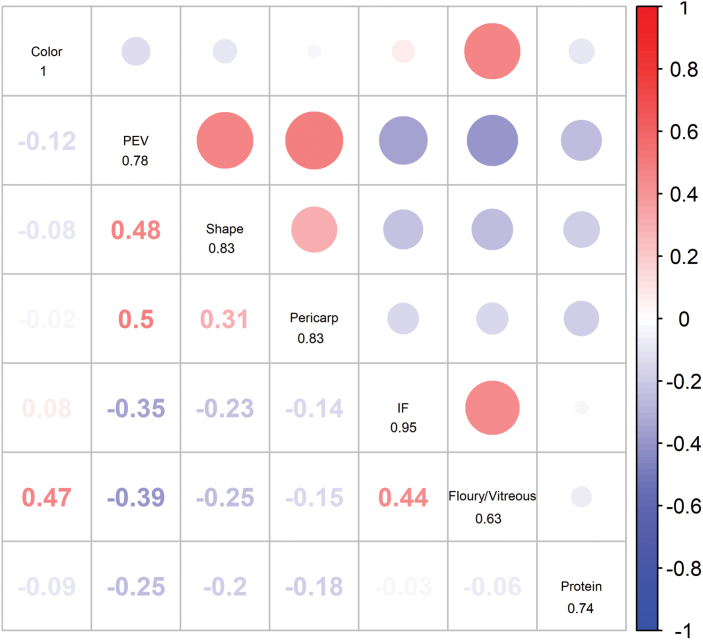
The pairwise correlations (Pearson’s *r*) among the seven traits based on the BLUP values. The value marked under each trait name in the diagonals was the heritability in broad sense for the corresponding trait. Correlations are represented as colored circles, blue indicating negative correlation, and red indicating positive correlation.

The correlations of BLUP values are presented in Fig. 2. Most traits were continuously and normally distributed, and hence presumed to exhibit quantitative inheritance (Supplementary Fig. S4). A wide range of phenotypes was observed, ranging from 50 ml to 680 ml for PEV, 0.28 to 4.01 for floury/vitreous, and 8.61–16.48% for protein (Table S4). A number of significant pairwise correlations were observed. For example, PEV was consistently, significantly, and positively correlated with pericarp (*r*=0.50, *P*<0.01) and popcorn shape (*r*=0.48, *P*<0.01), and significantly and negatively correlated with IF (*r*=–0.35, *P*<0.01), Floury/Vitreous (*r*=–0.39, *P*<0.01), and protein content (*r*=–0.25, *P*<0.01), confirming previously reported correlations between PEV and kernel quality traits (Vázquez-Carrillo *et al.*, 2019). Therefore, harder kernels, with a higher proportion of vitreous endosperm, and less protein content, will produce a greater expansion volume (PEV), more pericarp retention and a more “butterfly”-like shape. In addition, the broad-sense heritability (*H*^*2*^) of the seven popping-related traits ranged from 0.63 for floury/vitreous to 1 for color (Fig. 2), since the two replicates for color measurements were exactly the same.

### Genomic loci affecting popping-related traits in CIMMYT inbred lines

Each of the inbred lines was genotyped via cGBS, and SNPs were called using standard methodologies (see Methods). Subsequently, a GWAS was performed independently for each of the seven examined traits (i.e. color, PEV, shape, pericarp, IF, floury/vitreous, and protein content) using FarmCPU based on 155 083 SNPs (Supplementary Fig. S5). There was a significant peak on chromosome 6 for color with *P* value of 2.25E^-38^ (Table 2, Table S5, Fig. S5). The top SNP, S6_82019628, is a synonymous variant in *PSY1* (GRMZM2G300348), encoding a phytoene synthase. *PSY1* has been previously reported to reduce the carotenoid pigment content in maize endosperm, owing to a 378 bp InDel upstream of its transcription start site, and an SNP in its fifth exon that results in a Thr-to-Asn substitution (Fu *et al.*, 2013). This example highlights that our genotypic and phenotypic data can be analyzed via GWAS to yield functionally informative results about maize physiology.

**Table 2.** The identified SNPs associated with popping-related traits within annotated gene regions based on GWAS

Trait	SNP	Effect	*P*-value	Alleles	Annotation	GeneID	Gene Name	Description	Previous study
Color	S6_44382110	0.061	1.096E-09	T/A	synonymous_variant	GRMZM2G086294	zp15	Zein-beta Precursor (Zein-2)(16 kDa zein) (Zein clone 15A3)	
Color	S6_82019628	0.14	2.25E-38	A/G	synonymous_variant	GRMZM2G300348	y1	Phytoene synthase, chloroplastic Precursor (EC 2.5.1.32)	
Color	S6_86338050	0.038	1.33E-06	C/T	5_prime_UTR_variant	GRMZM2G082874	uwm3035	plant-specific domain TIGR01589 family protein	
Floury/Vitreous	S4_210389724	0.069	2.19E-06	G/A	intron_variant	GRMZM2G040559	uwm10774	tubulin alpha-6 chain	
PEV	S1_55451772	-15.95	2.34E-06	T/C	upstream_gene_variant	GRMZM2G056424	uwm46912	photosystem II 11 kD protein	qPEV1-1
PEV	S1_253277924	23.48	4.40E-06	A/T	upstream_gene_variant	GRMZM5G837732		CBL-interacting serine/threonine-protein kinase 15 (LOC100285495), mRNA	qPV1-3
PEV	S1_253277948	23.48	4.40E-06	C/T	upstream_gene_variant	GRMZM5G837732		CBL-interacting serine/threonine-protein kinase 15 (LOC100285495), mRNA	qPV1-3
PEV	S2_36136426	25.052	2.88E-06	C/T	intron_variant	GRMZM2G030598		kelch motif family protein	
PEV	S3_162805619	23.61	9.64E-06	C/T	downstream_gene_variant	GRMZM2G133398	vp1	Regulatory protein viviparous-1	
PEV	S3_213803780	-20.67	7.27E-06	T/C	splice_region_variant&intron_ variant	GRMZM2G047129		alpha-L-fucosidase 2	qBPV3-1
PEV	S5_175731057	29.74	1.14E-06	A/G	synonymous_variant	GRMZM2G024739	cry1	cryptochrome 1	
IF	S5_160833678	0.15	9.26E-06	A/C	synonymous_variant	GRMZM2G077989		RS21-C6 protein	
IF	S7_106078238	-0.26	9.63E-07	A/C	missense_variant	GRMZM2G046600	uwm17845	catalytic/ hydrolase	
IF	S9_153853384	-0.28	3.39E-06	G/A	upstream_gene_variant	GRMZM2G171466		nascent polypeptide-associated complex alpha subunit-like protein	
Protein	S7_165744712	-0.11	4.21E-06	C/A	upstream_gene_variant	GRMZM2G054032	uwm10642	F-box protein	
Shape	S5_190238066	-0.14	2.18E-06	T/C	upstream_gene_variant	GRMZM2G169558		protein kinase APK1A	
Shape	S8_136138158	0.14	2.97E-06	A/G	3_prime_UTR_variant	GRMZM2G100229		calmodulin binding protein	

**Fig. 3.**
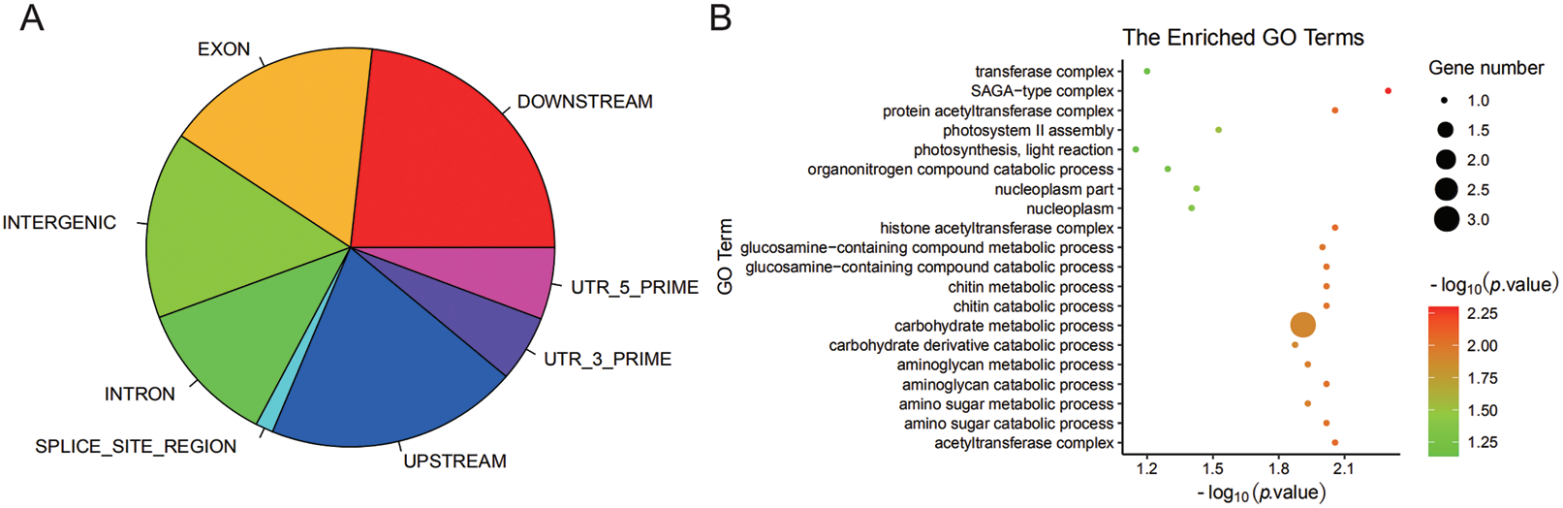
Gene annotation information and gene ontology for significant SNPs identified by GWAS. (A) Gene annotation; (B) gene ontology terms.

GWAS with these polymorphisms identified 162 SNPs distributed across the genome that were significantly (-log_10_*P*>5) associated with popping-related traits with *P*-values ranging from 9.88E^-06^ to 2.25E^-38^ (Supplementary Table S5). Fifteen SNPs related to PEV identified from the GWAS were found to be located within previously reported QTLs for popping expansion volume in maize (Table S5). For example, S1_55451772 was located at qPEV1-1 (Li *et al.*, 2008), S1_253277924 and S1_253277948 were in the region of qPV1-3 (Li *et al.*, 2007a, 2009), and S3_213803780 was within qBPV3-1 (Li *et al.*, 2009). Of the 162 significant SNPs, 15% were annotated in intergenic regions, 43% SNPs were in the upstream and downstream regions, 12% SNPs were in the intron regions, and 30% were in coding regions, splice sites, 3’UTR, and 5’ UTR regions (Table S5, Fig. 3A). In total, 118 known genes were mapped by the significant SNPs. Following Gene Ontology analysis we found that most genes are involved in metabolic and biosynthetic processes (Table S5, Fig. 3B). The functions of most of these genes (*n*=101) have not been defined (Table S5). Therefore, the functional annotations of the remaining 17 are displayed in Table 2. For example, *ZP15*, related to color, is a zein-beta precursor; *UWM46912* encodes a photosystem II 11 kDa protein; regulatory protein viviparous-1 (*VP1*) and cryptochrome 1 (*CRY1*) affect PEV; and *UWM10642*, which encodes an F-box protein, is associated with protein content (Table 2).

Pleiotropic genes play important roles in understanding correlations among phenotypes (Chen and Lübberstedt, 2010), but loci simultaneously controlling multiple popping-related traits have seldom been reported (Li *et al.*, 2008). To dissect the genetic architecture of the correlations across different traits, we analyzed the association networks among the seven examined traits. We found that floury/vitreous and pericarp shared a common significant association, SNP S7_82404867 (Supplementary Table S5). This finding was consistent with the correlation pattern of the traits, as shown in Fig. 2: a significant correlation was observed between PEV and pericarp (*r*=0.5, *P*<0.01), PEV and protein (*r*=–0.25, *P*<0.01), and floury/vitreous and pericarp (*r*=–0.15, *P*<0.01). PEV was connected to pericarp and protein content by SNPs S2_96341835 and S2_217692527. These results suggest that the popping-related traits might be genetically co-regulated.

**Fig. 4.**
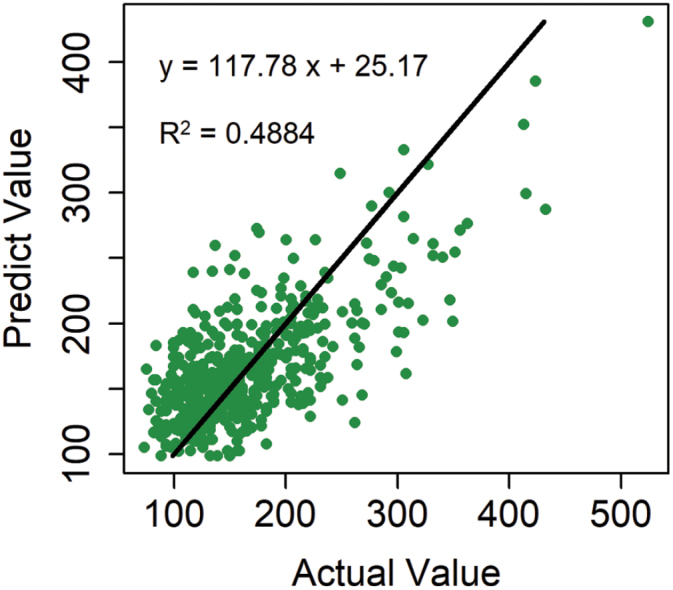
Prediction of the observed phenotype of PEV (popping expansion volume) by the significant SNPs associated with PEV. The predicted value indicates the performance of the PEV predicted by identifed QTL, and observed value indicates the phenotype measured.

To better understand the popping-related traits, we estimated the proportion of the phenotypic variance explained by significant SNPs, which averaged 43% across the seven traits, ranging from 28–83% (Fig. 4 and Supplementary Fig. S6). For the most important trait, PEV, 58 associated signals explained 48.84% of the phenotypic variance. For color, 26 significant loci explained 83% of the phenotypic variance. For the other five traits, associated SNPs explained less than half of the total observed phenotypic variance (Fig. S6).

### Identification of popping-related loci

**Fig. 5.**
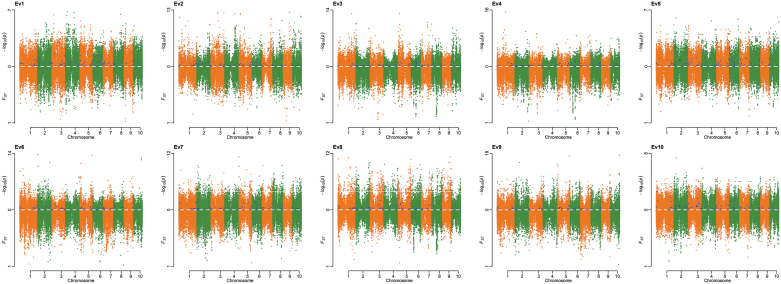
Miami plots from EigenGWAS. The upper panels show data for –log(*P*_GC_) and lower panels, for *F*_st_ for Ev1-Ev10, based on 526 CIMMYT inbred lines with 155 083 SNPs. Dots in blue are associated with seven popping-related traits, with their detailed information listed in Table S5.

**Fig. 6.**
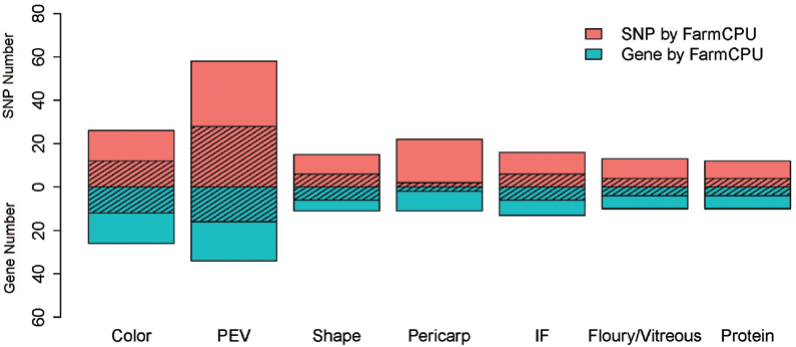
The SNPs and gene number identified by GWAS and EigenGWAS for seven traits.

**Fig. 7.**
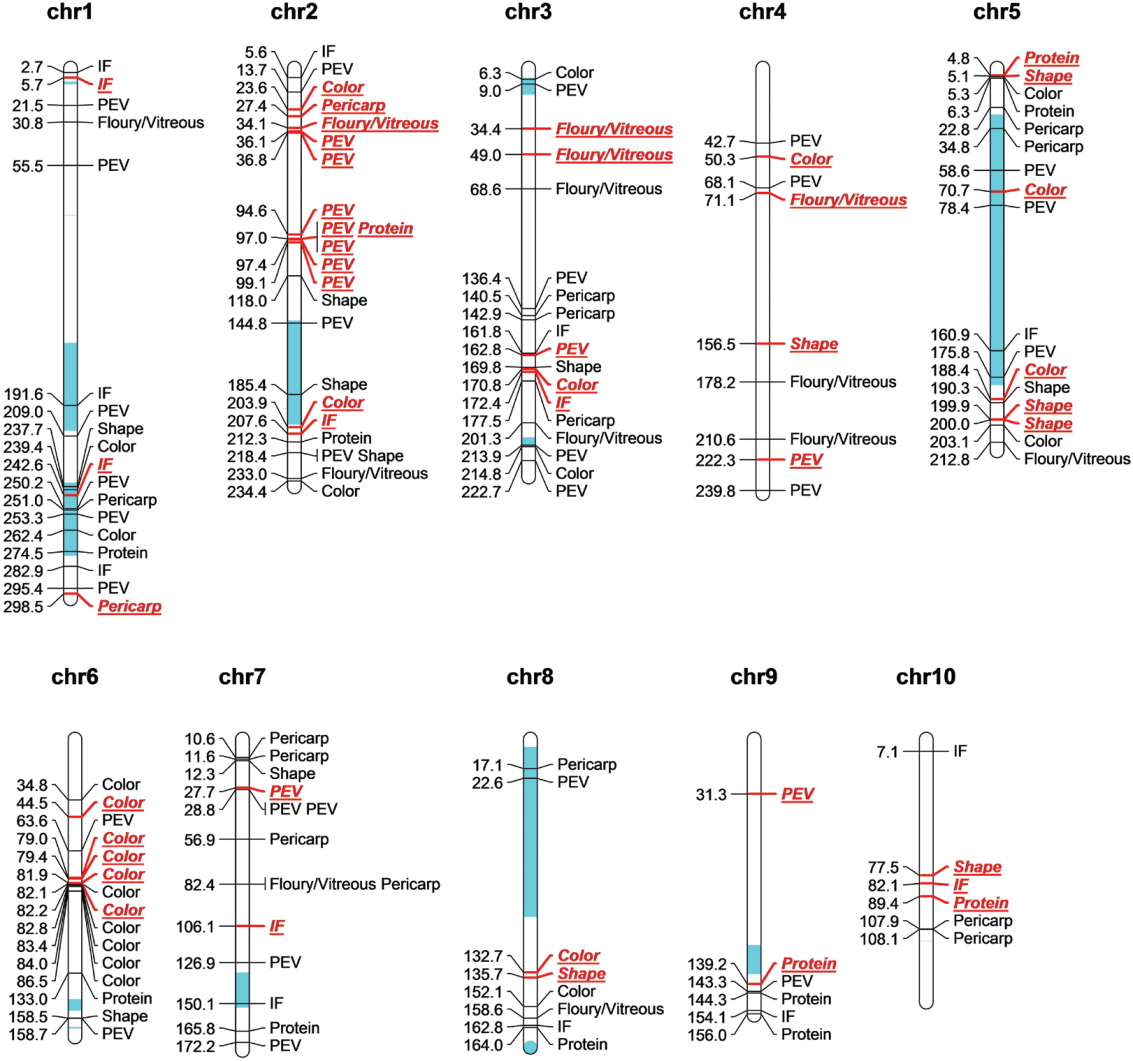
Distribution of 162 significant SNPs for seven popping-related traits. For each SNP, its physical position (Mb) and related trait name are shown on the two sides of its located chromosome. Previously reported regions that associated with PEV are indicated in blue. Loci with trait names in red and italics have undergone selection.

**Fig. 8.**
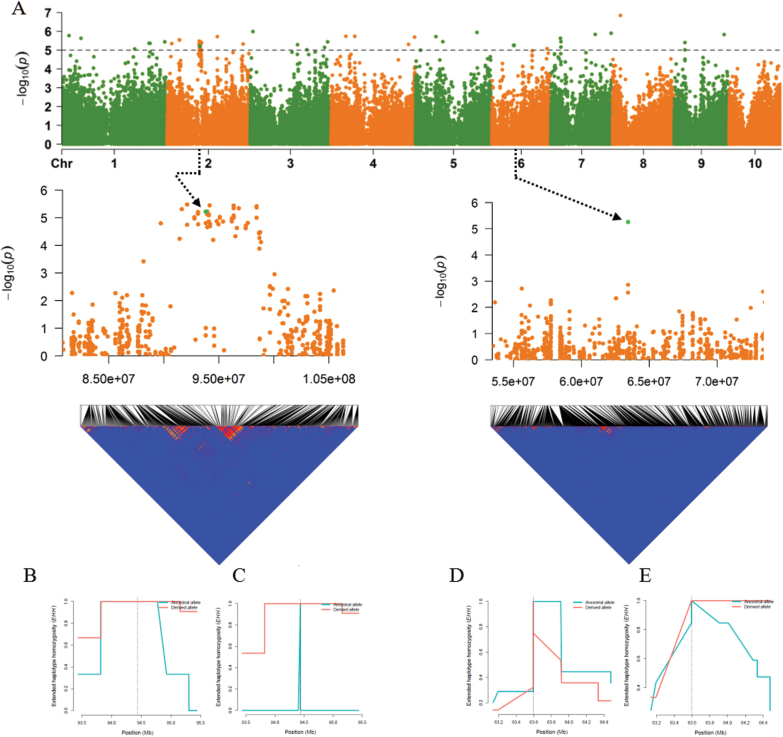
Genome-wide Manhattan plot and linkage disequilibrium (LD) blocks. Data are from S2_93806542 in the region from 70 to 90 Mb on chromosome 2, and S6_63434708 in the region from 80 to 100 Mb on chromosome 6. The horizontal grey dashed line indicates genome-wide significant thresholds -log10 (*p*) value of 5 (A), and EHH (extended haplotype homozygosity) for intervals in the length of 20 Mb with S2_93806542 and S6_63434708 in the middle when selecting 100 lowest PEV (B and D) and 100 highest PEV (C and E) accessions in 764 landrace populations.

EigenGWAS was conducted to examine the selection loci for 155 083 SNPs based on 526 CMLs for the top ten largest eigenvectors (Fig. 5). The mean of genetic relatedness across CMLs was –0.0018, indicating that the effective sample number was 547.76 for the collection, and the effective number of genome segments was 651.34. As a result, we identified 60 677 loci (top 5%) under selection, distributed on 10 chromosomes, with the highest number of significant loci (i.e. 11 211 SNPs) on chromosome 1. Of 162 popping-related loci, 62 were identified to have undergone selection (Figs 6–8). PEV had the most significant loci with 28 under selection, with six for shape and two for pericarp (Figs 6, 7). These results indicated that most of the popping-related loci had undergone selection. Distribution of the SNPs varied considerably across different traits, with PEV having the highest numbers of SNPs (*n*=58), and protein content, the lowest (*n*=12 SNPs; Figs 6, 7). Of the 48.84% of the phenotypic variation explained by QTLs controlling PEV (Fig. 4), 20.90% could be explained by SNPs associated with PEV, exhibiting evidence of selection (Supplementary Fig. S7). For color, this proportion is two-thirds (61.44% of 83.19%), around half for shape (16.09% of 27.64%), 37% for protein (12.59% of 34.13%), a quarter for IF (10.97% of 44.37%), and less than 10% for pericarp and floury/vitreous (Fig. 4, Fig. S7). To evaluate the variation for alleles that promote popping in non-popcorn CML inbreds, we calculated the accumulated frequencies of favorable alleles that promote popping for each CML. The results showed that on average, CMLs contain 55.27% of the alleles that promote popping, with 507 lines harboring over 50% of the alleles (Fig. S8).

### Validation of popping-related loci in tropical landraces

To validate the popping-related loci, EHH analysis of two PEV-related loci (S2_93806542 and S6_63434708) was conducted in the set of 764 landrace populations. We narrowed down the candidate region on chromosome 2 to a 7 Mb region with 16 genes from 91 435 963 bp to 98 827 228 bp by linkage disequilibrium analysis (Fig. 8A). We evaluated the EHH values for 100 accessions with the largest PEV and 100 accessions with the lowest PEV (Supplementary Table S2) EHH tests showed that the S2_93806542-A haplotypes extended further than the reference S2_93806542-T haplotypes, depicting the long-range haplotype homozygosity across the region of S2_93806542-A haplotypes. Large differences in PEV were observed between the two tails (100 accessions each) of the 764 landraces for the S2_93806542-A haplotype and the S2_93806542-T haplotype groups (Fig. 8B, C). Similarly, there were large differences in PEV between the S6_63434708-C and S6_63434708-A haplotypes in the same 100 top and bottom ranking accessions (Fig. 8D, E). These data from a separate set of populations validate the associations observed between the SNPs and PEV in the CMLs.

## Discussion

### A large scale genetic study on popping-related traits by whole genome sequencing

At present, genetic studies of popping-related traits are limited to conventional SSR markers. For example, Lu *et al.* (2003) used 259 SSR markers and 160 selfed plants of the backcrossing families (BC_1_S_1_) derived from popcorn and dent maize parents, and detected four QTLs related to PEV on chromosomes 1, 3, and 5. Using a total of 101 SSR markers to detect QTLs in 194 F_3_ plants derived from the popcorn inbred A-1–6 and flint maize inbred V273, Babu *et al.* (2006) detected four, four, and five QTLs related to popping rate, PEV, and unpopped kernel rate, respectively. From 2007 to 2009, 10 QTLs associated with PEV, eight QTLs related to flake size, and seven QTLs associated with popping rate, were identified by 193 SSR markers using the 259 F_2:3_ and 220 BC_2_F_2_ derived from popcorn inbred N04 and dent corn inbred Dan232 (Li *et al.*, 2007a, b, 2008, 2009; Supplementary Table S1). As we know, SSRs are normally non-genic markers, while many SNPs are intragenic (Shi *et al.*, 2013). Therefore, the genomic loci identified by cGBS are more likely to represent variations in the causal genes of popping traits. As a result of our study, 162 popping-related loci were identified for seven popping-related traits by cGBS, which comprehensively incorporated most of the previously known quantitative loci for popping expansion volume (Fig. 7, Table S5). A large proportion of the phenotypic variation was explained by the identified SNPs associated with PEV (Figs 4, S6), which indicates that the additive model can accurately predict PEV. In view of this result, it is helpful to improve the accuracy of prediction and accelerate the breeding cycle by incorporating numerous small-effect QTLs in genomic selection. A synergy among popping-related traits was also uncovered, which indicates that the popping characteristic is a complex trait, determined by many factors. Due to the different sequencing platforms and versions of the reference genome employed in this study, of the 162 popping SNPs identified by the cGBS platform using B73 AGPv2 as a reference genome, only five loci could be projected to the tGBS platform using B73 AGPv3 as reference genome, and only two out of five loci could be validated in the set of 764 tropical landrace populations. The deciphered and validated genetic architecture of popping-related traits will provide a valuable resource for future molecular physiological studies and for popcorn breeding programs.

### Kernel traits underwent selection during maize improvement

To further understand the history and spread of maize, Ramos-Madrigal *et al.* (2016) characterized the draft genome of a 5310-year-old archaeological cob found during excavations in the Tehuacan Valley of Mexico. Many genes associated with key domestication traits, such as *teosinte glume architecture 1* (*TGA1*)*, zea agamous-like1* (*ZAGL1*)*, sugary1* (*SU1*), and *waxy 1* (*WX1*), existed in the ancestral state, sharply contrasting with the ubiquity of derived alleles in extant landraces. Their findings suggest that much of the evolution during domestication has been gradual, and they encourage further paleogenomic research to address provocative questions about the world’s most productive cereal. The most ancient form of maize was a popcorn (*Zea mays* L. var. *Everta*), and as such it is an ideal material for researchers to access a vast archive of paleogenomic data, permitting detailed investigations of prehistoric, as well as recent, selective pressures, and ultimately achieving a new understanding of the formation of maize landraces and improved maize lines. 

Recently, Li *et al.* (2019) showed that the domestication process of maize was at least partly driven by environmental parameters. These included soil pH at 5 cm depth, and rainfall in 1143 maize accessions from diverse ecological adaptation zones, including tropical lowland, highland, sub-tropical/mid-altitude, and temperate, covering major ecotypes of maize resources developed during domestication and breeding. Due to the lack of replicated quantitative measurements of popping characteristics for these 1143 accessions, two groups (popcorn versus non-popcorn) were identified, and these qualitative phenotypes were used to map the popping-related loci and test if they had undergone selection (Li *et al.*, 2019). With this very limited description of phenotypic variation of popping performance, Li *et al.* (2019) failed to find popping-related loci under selection. 

To improve the analysis, in this study a comprehensive, quantitative measurement of kernel traits was performed. As a result, we were able to identify 162 SNPs associated with the kernel traits that we measured, of which 62 (38%) exhibit evidence of having been under selection. For seven traits, more than 30% of the significant SNPs exhibit evidence of selection, except for the percentage of pericarps. Those traits (and the percentage of SNPs) are color (46%), PEV (48%), shape (40%), IF (38%), protein (33%), and floury/vitreous (31%; Fig. 6). Given the effects of environmental and climate factors on maize adaptation (Li *et al.*, 2019), a different number of selection loci has been detected on seven traits. The reason is perhaps due to the varying selection pressures presented by the environment and climate change, and different preferences of different human groups. In total, 42–66% of the alleles for kernel traits that promote popping were found across CMLs (Supplementary Fig. S8). It suggests that selection of popping characteristics was gradual, which is consistent with the findings of Ramos-Madrigal *et al.* (2016) for other traits. The results shown in this study, therefore, provide a foundation for further paleogenomic and molecular biology research and pinpoint the importance of environment and climate for popcorn adaptation.

## Perspectives

In this study, the 162 identified loci that we identified incorporated all of the previously known loci associated with popping-related traits. Beyond that, 147 loci were reported here for the first time. We discovered that non-popcorn inbreds (CMLs) contain variation for alleles that promote popping. Thus, our study represents a new benchmark for genetics in popcorn, and moving forward, should be helpful in dissecting both evolutionary and functional hypotheses. Specifically, it will guide efforts to determine the genetic basis of popcorn physiology, evolution, and history, assist gene discovery and breeding, and provide a more detailed understanding of the role of gradual popcorn domestication during the evolution of teosinte–to–popcorn-to-diversified modern maize. Genome sequences contain all kinds of information that control and influence biological functions. In this study, genetic variation was aligned to the B73 reference genome which has its own unique selection history. The development of the other gold-standard temperate maize genomes, Mo17 (Sun *et al.*, 2018) and W22 (Springer *et al.*, 2018), and especially the tropical maize genome SK (Yang *et al.*, 2019) and quality protein maize (QPM) maize genome K0326Y (Li *et al.*, 2020), provide valuable resources for us to increase the proportion of potentially useful genomic variation, and to characterize the phenotypic variation of popping-related traits in a more comprehensive framework in the near future.

## Supplementary Data

The following supplementary data are available at *JXB* online.

Fig. S1. The SNP density of 155 083 SNPs on 10 chromosomes.

Fig. S2. Genome-wide average linkage disequilibrium (LD) decay over physical distance. 

Fig. S3. Principal component analysis (PCA) of 155 083 SNPs based on 526 CMLs for GWAS.

Fig. S4. The phenotypic distribution of the seven traits. 

Fig. S5. Manhattan plots and QQ (Quantile-Quantile) plots for six traits. 

Fig. S6. Prediction of the observed performance for six traits using the significant SNPs associated with the corresponding traits. 

Fig. S7. Prediction of the observed performance for seven traits using the common significant SNPs identified by GWAS and EigenGWAS. 

Fig. S8. The frequency distribution of popping-related SNPs that promote popping in CMLs.

Table S1. Summary of the popping-related loci identified in previous studies.

Table S2. The information on the seven traits measured in two replications for 526 CMLs.

Table S3. The information and phenotype for the 764 maize tropical landrace populations.

Table S4. Estimates of variance components for six traits in this study.

Table S5. The significant SNPs associated with popping-related traits in 526 CMLs.

eraa480_suppl_Supplementary_Table

eraa480_suppl_Supplementary_Figure

## Data Availability

The data supporting the findings of this study are available from The Global Popcorn Project http://hdl.handle.net/11529/10548274.

## Abbreviations

BLUP: Best linear unbiased prediction
cGBS: conventional genotyping-by-sequencing
CIMMYT: The International Maize and Wheat Improvement Center
CML: *C*IMMYT *M*aize *L*ine
EHH: Extended haplotype homozygosity
GWAS: Genome-wide association study
IF: Flotation index
LD: Linkage disequilibrium
MAF: Minor allele frequency
o2: opaque-2
PEV: Popping expansion volume
tGBS: tunable genotyping-by-sequencing
