# Supplementary material for: Genome-wide analyses reveal footprints of divergent selection and popping-related traits in CIMMYT’s maize inbred lines
Source: J Exp Bot. 2020 Oct 18;72(4):1307–20. doi: 10.1093/jxb/eraa480 (PMC7904155; doi:10.1093/jxb/eraa480)

Fig. S1 The SNP density of 155,083 SNPs on 10 chromosomes.

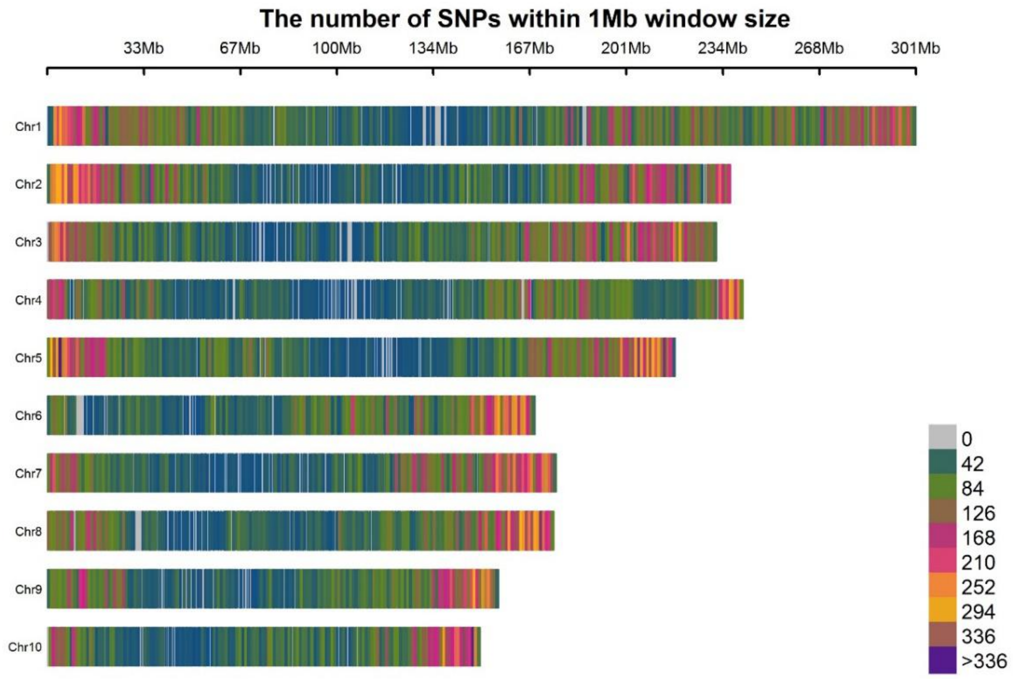

Fig. S2 Genome-wide average linkage disequilibrium (LD) decay over physical distance. Plots of pair-wise single-nucleotide polymorphism LD r^2 values as a function of inter-marker map distance (kb).

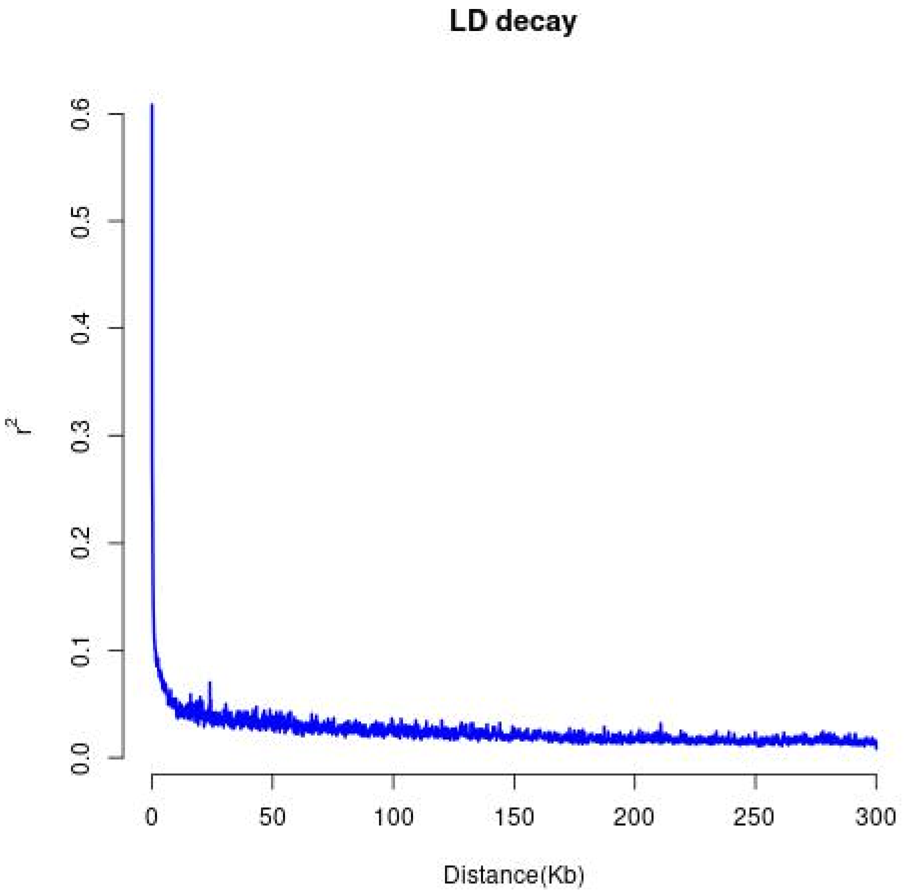

Fig. S3 Principal component analysis (PCA) of 155,083 SNPs based on 526 CMLs for GWAS.

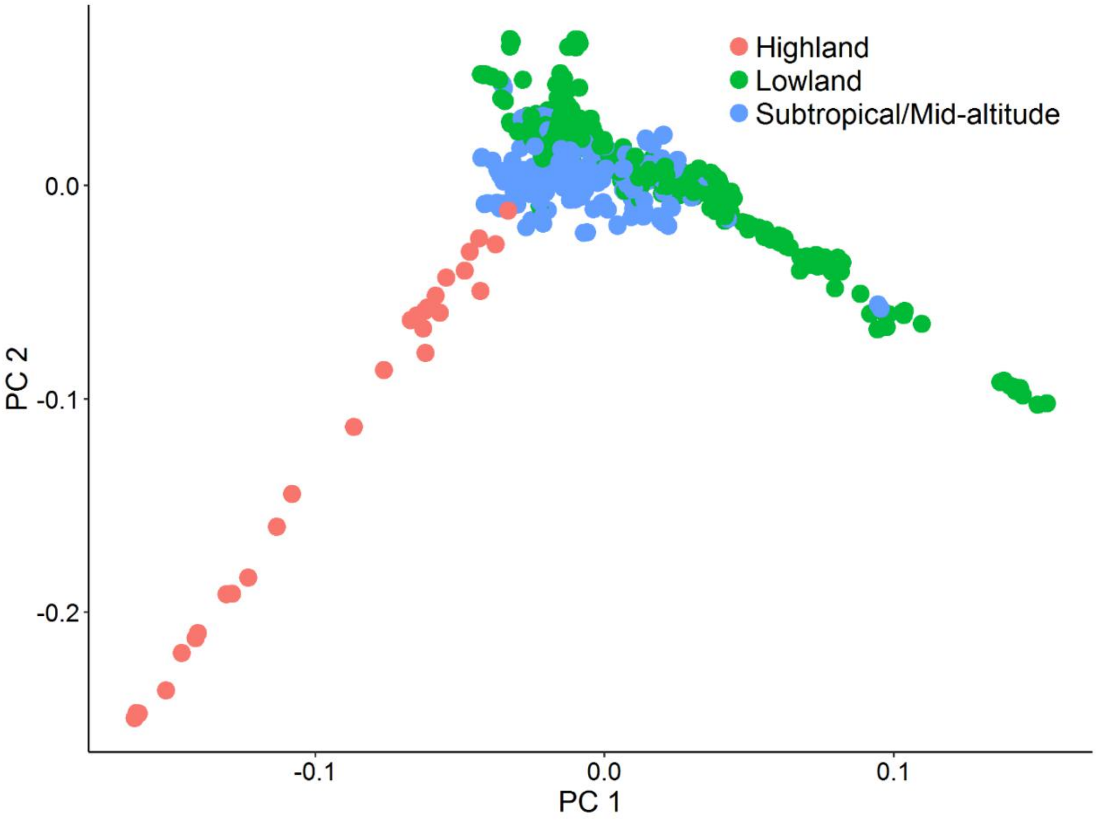

Fig. S4 The phenotypic distribution of the seven traits. A is for color; B is for PEV; C is for shape; D is for pericarp; E is for IF, F is for floury/vitreous; and G is for protein. See Table 1 for descriptions of traits.

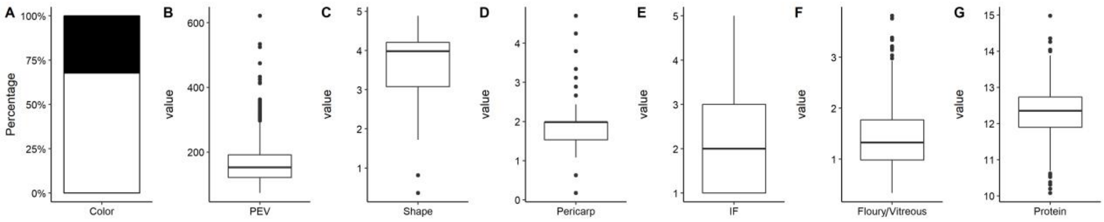

Fig. S5 Manhattan plots and QQ (Quantile-Quantile) plots for six traits, the horizontal grey dashed line indicate genome-wide significant thresholds -log10 (*p*) value of 5. See Table 1 for descriptions of traits.

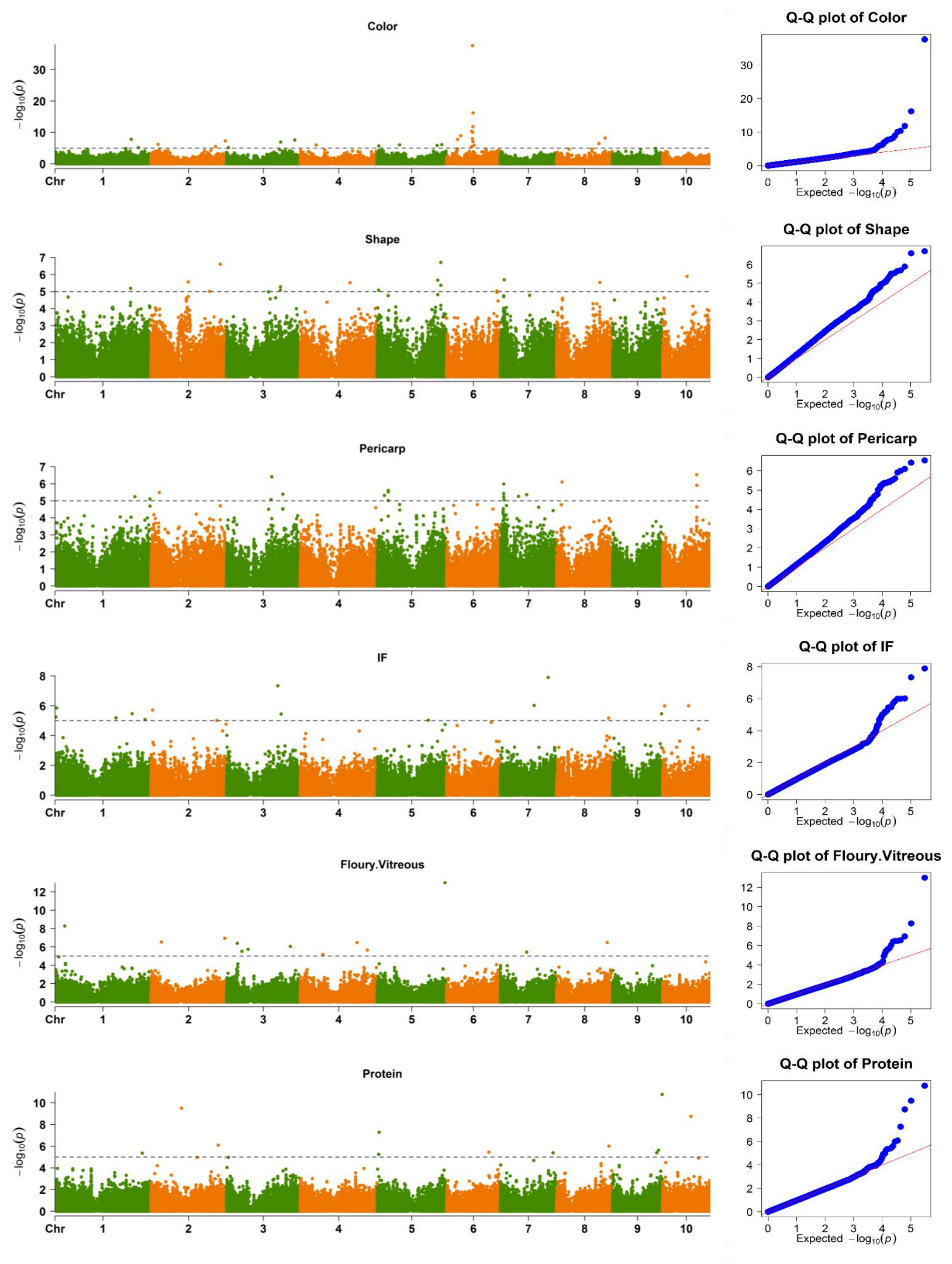

Fig. S6 Prediction of the observed performance for six traits using the significant SNPs associated with the corresponding traits. See Table 1 for descriptions of traits.

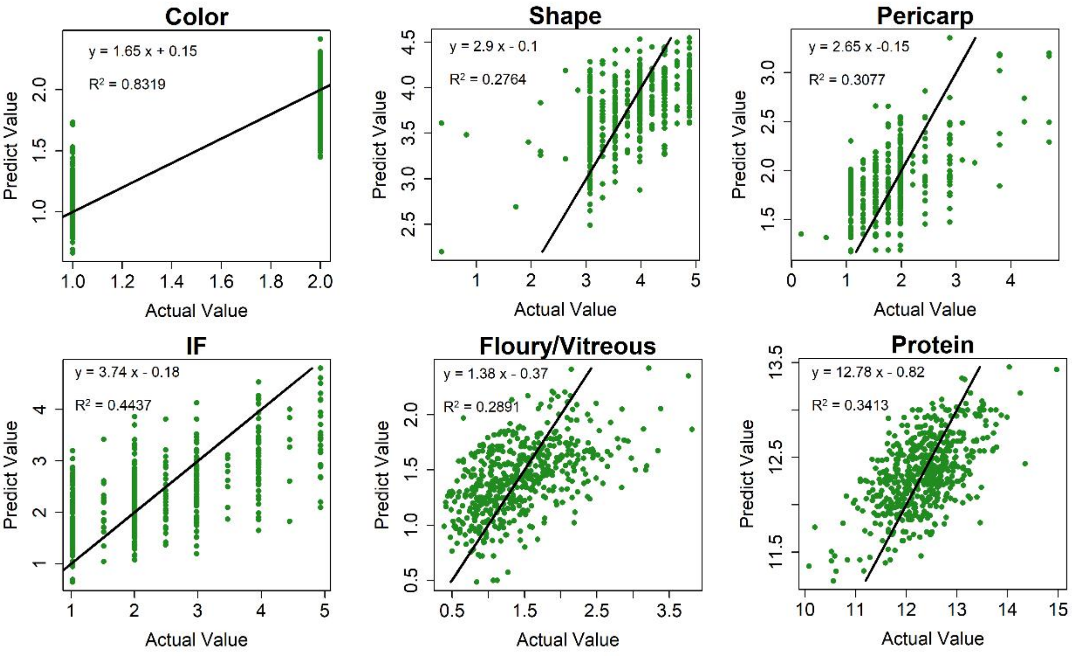

Fig. S7 Prediction of the observed performance for seven traits using the common significant SNPs identified by GWAS and EigenGWAS. See Table 1 for descriptions of traits.

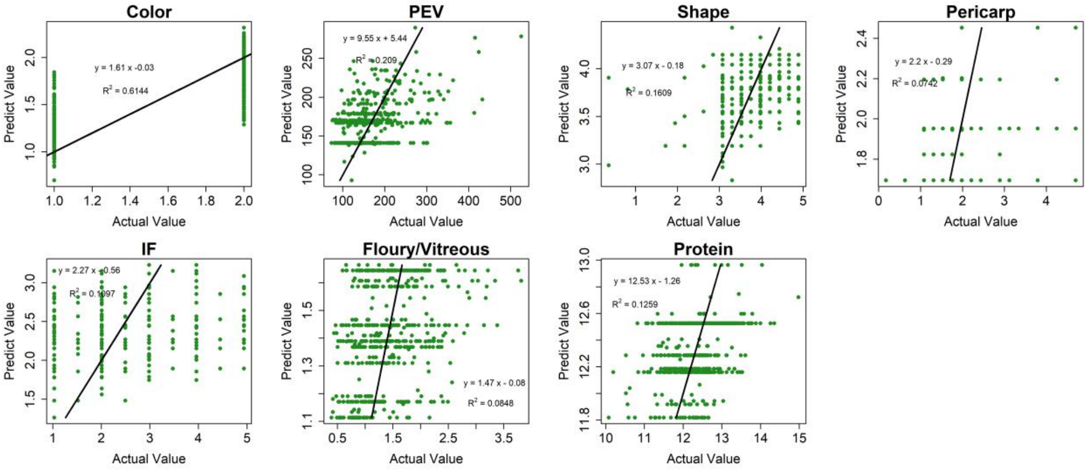

Fig. S8 The frequency distribution of popping related SNPs that promote popping in CMLs..

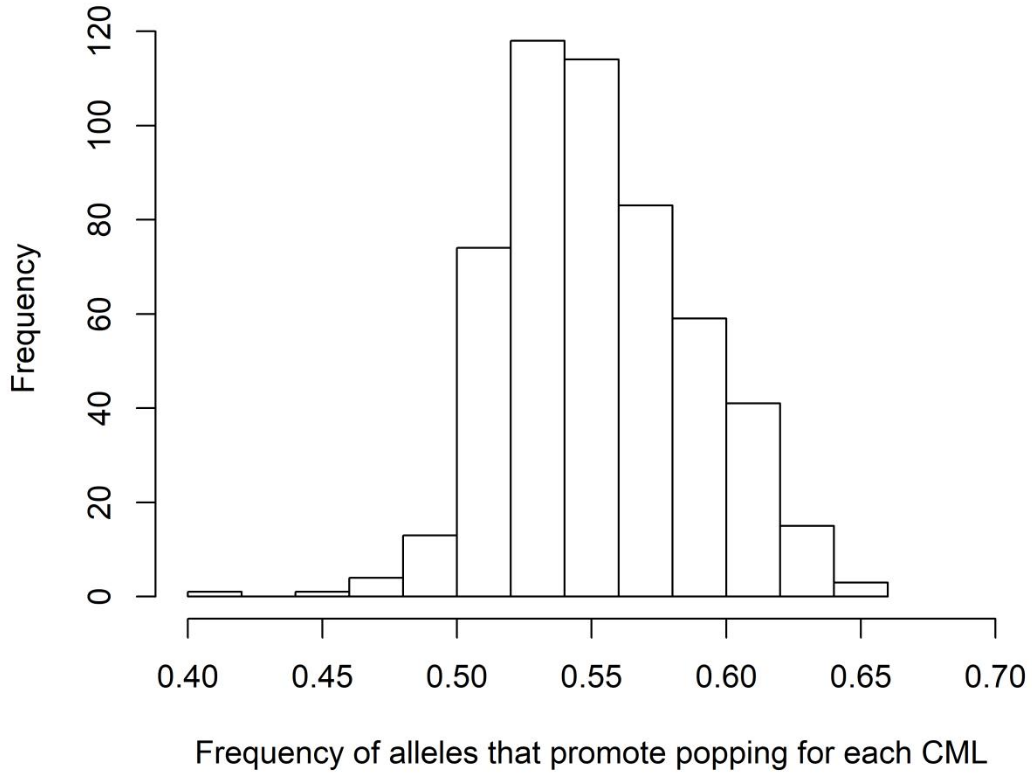

Supplement: eraa480_suppl_Supplementary_Figure [file eraa480_suppl_supplementary_figure.pdf]
